# A Review of Shannon and Differential Entropy Rate Estimation

**DOI:** 10.3390/e23081046

**Published:** 2021-08-13

**Authors:** Andrew Feutrill, Matthew Roughan

**Affiliations:** 1 CSIRO/Data61, 13 Kintore Avenue, Adelaide, SA 5000, Australia; 2 School of Mathematical Sciences, The University of Adelaide, Adelaide, SA 5005, Australia; matthew.roughan@adelaide.edu.au; 3 ARC Centre of Excellence for Mathematical & Statistical Frontiers, The University of Melbourne, Parkville, VIC 3010, Australia

**Keywords:** entropy rate, estimation, parametric, non-parametric

## Abstract

In this paper, we present a review of Shannon and differential entropy rate estimation techniques. Entropy rate, which measures the average information gain from a stochastic process, is a measure of uncertainty and complexity of a stochastic process. We discuss the estimation of entropy rate from empirical data, and review both parametric and non-parametric techniques. We look at many different assumptions on properties of the processes for parametric processes, in particular focussing on Markov and Gaussian assumptions. Non-parametric estimation relies on limit theorems which involve the entropy rate from observations, and to discuss these, we introduce some theory and the practical implementations of estimators of this type.

## 1. Introduction

The estimation of the entropy of a random variable has long been an area of interest in Information Theory. From the original definition by Shannon [1], the interest in development of information theory and entropy as a concept was motivated by aiming to understand the uncertainty of sources of information and in the development of communication theory. In real systems, understanding this uncertainty allows more robust models and a better understanding of complex phenomena.

Estimation of the entropy of random variables has been reviewed on several occasions, with reviews that have covered the estimation of Shannon, differential, and other types of entropy measures. A recent survey by Verdu [2] reviewed techniques for empirical estimation of many information measures, such as entropy, relative entropy and mutual information, for both discrete and continuous data. Amigó et al. [3] surveyed generalised entropies, for further quantification of complexity and uncertainty of random variables. Rodriguez et al. [4] surveyed and reviewed the performance of 18 entropy estimators for short samples of data, assessing them on their bias and mean squared error. A comparison of different generalised entropy measures, and their performance, was recently performed by Al-Babtain et al. [5].

In this paper, we review techniques for estimating the entropy rate, a measure of uncertainty for stochastic processes. This is a measure of the average uncertainty of a stochastic process, when measured per sample. Shannon’s initial work considered the problem of quantifying the uncertainty of Markov sources of information [1]. We will be considering estimation techniques of the entropy rate for both discrete and continuous data, therefore covering both Shannon and differential entropy rate estimation.

There are two main estimation paradigms that are used in statistical estimation, parametric and non-parametric estimation. Parametric techniques assume a model for stochastic process that generates the data, and fitting parameters to the model [6]. In many cases, these parameters are estimated and then used directly in an entropy rate expression, which we call plug-in estimation. Non-parametric approaches, on the other hand, make very few assumptions on the process that generates the data. However, they can contain assumption properties, such as stationarity [6]. Fewer assumptions for an estimator can lead to more robustness. This review will cover techniques using both of these approaches, outlining what assumptions are used in the generation of the estimates. We categorise the references used into parametric and non-parametric entropy rate estimates and modelling estimates in Table 1. We review estimation techniques for continuous and discrete-valued data, and continuous and discrete-time data, the references in this paper are categorised by these properties in Table 2.

The parametric estimation techniques reviewed model the data as Gaussian processes, Markov processes, hidden Markov models and renewal processes. For Gaussian processes, due to the equivalence of entropy rate estimation and spectral density estimation, which we discuss below, we introduce some literature on spectral density estimation, such as maximum entropy and maximum likelihood techniques.

Non-parametric estimators are often based on limit theorems of an expression of the entropy rate, with estimation being made on a finite set of data. We review and present assumptions and properties of non-parametric entropy rate estimation techniques for Shannon entropy, which are based on limit theorems of string matches. For differential entropy rate estimation, we present three techniques that were developed as measures of complexity of time series, rather than strictly as entropy rate estimators. However, in some special cases, such as first order Markov chains, they have been shown to converge to the entropy rate, and therefore, in practice, have been used as entropy rate estimators. Then, we present another approach using conditional entropy estimates, based on observations of a finite past, that provides an exact estimate, given some assumptions. There are far fewer techniques that have been developed for continuous-valued random variables, which is not surprising given the history of development of information theory for transmission of data.

## 2. Entropy and Entropy Rate

The objects of interest here are the Shannon entropy rate for discrete valued processes, and the differential entropy rate for continuous valued processes. For the sake of precision, we provide the definitions we will use in the review.

**Definition 1.**  *Given a collection of random variables, $X_{1},\ldots ,X_{n}$, with support on $\Omega_{1},\ldots ,\Omega_{n}$, respectively, the joint entropy of the collection of discrete random variables is,*

$$\begin{array}{cc} H(X_{1},\ldots ,X_{n}) & =-\sum_{x_{1}\in \Omega_{1}}\ldots \sum_{x_{n}\in \Omega_{n}}p\left(x_{1},\ldots ,x_{n}\right)\log p\left(x_{1},\ldots ,x_{n}\right). \end{array}$$

For discrete-time stochastic processes, the entropy rate is an asymptotic measure of the average uncertainty of a random variable.

**Definition 2.**  *For a discrete-valued, discrete-time stochastic process, $\chi ={\left\{X_{i}\right\}}_{i\in N}$, the entropy rate is defined as,*

$$\begin{array}{cc} H(\chi ) & =\lim_{n\to \infty }\frac{1}{n}H\left(X_{1},\ldots ,X_{n}\right). \end{array}$$

We define the joint entropy similarly to the discrete random variable case.

**Definition 3.**  *The joint entropy of a collection of continuous random variables, $X_{1},\ldots ,X_{n}$, with support on $\Omega_{1}\times \ldots \times \Omega_{n}$, with a joint density function, $f(x_{1},\ldots ,x_{n})$, is*

$$\begin{array}{cc} h(X_{1},\ldots ,X_{n}) & =-\int_{\Omega_{1}}\ldots \int_{\Omega_{n}}f\left(x_{1},\ldots ,x_{n}\right)\log f\left(x_{1},\ldots ,x_{n}\right)dx_{1}\ldots dx_{n}. \end{array}$$

Then, we can define the differential entropy rate similarly to the Shannon entropy rate.

**Definition 4.**  *The differential entropy rate for a continuous-valued, discrete-time stochastic process, $\chi ={\left\{X_{i}\right\}}_{i\in N}$, is defined as,*

$$\begin{array}{cc} h(\chi ) & =\lim_{n\to \infty }\frac{1}{n}h\left(X_{1},\ldots ,X_{n}\right). \end{array}$$

The entropy rates H(χ) and h(χ) are the quantities that we wish to estimate.

There are other entropic measures which are used to quantify uncertainty. For example, the Rényi entropy [57], and its associated entropy rate, which is a generalisation of the Shannon entropy. Another approach to quantifying the uncertainty of stochastic processes is through an extension of a relative measure, mutual information, and the limit as a stochastic process, the mutual information rate [58]. However, we are not considering any generalised entropy quantities in this work.

A helpful property in the analysis of stochastic processes is stationarity, which expresses the idea that the properties of the process do not vary with time.

**Definition 5.**  *A stochastic process, $\chi ={\left\{X_{i}\right\}}_{i\in N}$, with a cumulative distribution function for its joint distribution at times $t_{1}+\tau ,\ldots ,t_{n}+\tau$, of $F_{X}\left(t_{1}+\tau ,\ldots ,t_{n}+\tau \right)$. We say that the process is stationary if and only if $F_{X}\left(t_{1}+\tau ,\ldots ,t_{n}+\tau \right)=F_{X}\left(t_{1},\ldots ,t_{n}\right),\forall \tau ,t_{1},\ldots ,t_{n}\in R,\forall n\in N$.*

This leads to another characterisation of the entropy rate for stationary processes, which is based on the limit of the conditional entropy.

**Definition 6.**  *For random variables, X and Y such that $\left(X,Y\right)∼p\left(x,y\right)$, the conditional entropy is defined as*

$$\begin{array}{cc} H(Y|X) & =-\sum_{x\in \Omega }\sum_{y\in \Omega }p\left(x,y\right)\log p\left(y|x\right). \end{array}$$

The following theorem gives a helpful way to analyse the entropy rate for stationary processes, and has been used in the development of estimation techniques [54].

**Theorem 1.**  *For a stationary stochastic process, the entropy rate exists and is equal to*

$$\begin{array}{cc} H(\chi ) & =\lim_{n\to \infty }H\left(X_{n}|X_{n-1},\ldots ,X_{1}\right). \end{array}$$

Briefly we introduce the estimation problem. Given a sample of data, $x_{1},\ldots ,x_{n}$, we are aiming to define a function, $T_{n}$, such that we generate an estimate of a quantity, θ, as $\hat{\theta }=T\left(x_{1},\ldots ,x_{n}\right)=T_{n}$. We will be assessing the quality with respect to some properties of estimators, which we will describe here. The first property *consistency* means that an estimator converges to the true value of the parameter being estimated as the number of data tends to infinity ([59], p. 234). Secondly, we discuss asymptotic normality, which means that the estimates are normally distributed, with variance that is related to the number of data ([59], p. 243). The third property we discuss is efficiency, which measures the variance of the estimation technique with respect to the lowest possible variance ([59], p. 250), the reciprocal of the Fisher information, as defined by the Cramer–Rao bound ([60], p. 480). We also discuss the bias, which measures if the expectation of the estimator differs from the true value ([59], p. 120). The mean squared error is used to quantify the quality of the estimate, by the squared differences between the data and estimates ([59], p. 121).

## 3. Parametric Approaches

In this section, we will discuss parametric approaches to estimate the entropy rate of a process from observed data. Parametric estimators assume a model for the data, estimate some aspects of the model from the data and then directly calculate the entropy rate from those estimates. The three model types that are Gaussian processes, Markov processes, and renewal/point processes.

### 3.1. Gaussian Processes

First, we will cover a class of processes that are defined by the assumption that the finite dimensional distributions are normally distributed. These are called Gaussian processes, and are often used to model real data. Gaussian processes, as an extension of normally distributed random variables, are completely characterised by their first and second statistics ([61], p. 28). Since the spectral density is the Fourier transform of the autocovariance, all the information for the process is encoded in the spectral density.

**Definition 7.**  *A stochastic process is called a Gaussian process if and only if every finite collection of random variables from the stochastic process has a multivariate Gaussian distribution. That is, for every $t_{1},\ldots ,t_{k}\in R$,*

$$\begin{array}{c} \left(X_{t_{1}},\ldots ,X_{t_{k}}\right)∼N\left(\mu ,\sum \right), \end{array}$$

*where μ is the vector of expected values and ***Σ*** is the covariance matrix.*

The entropy rate of a Gaussian process is given by,

(1) $$h\left(\chi \right)=\frac{1}{2}\log\left(2\pi e\right)+\frac{1}{4\pi }\int_{-\pi }^{\pi }\log\left(f\left(\lambda \right)\right)d\lambda ,$$

where f(λ) is the spectral density of the process ([62], p. 417).

This reduces the estimation task down to estimating the spectral density of the process. That is, using an approach to create an estimate of the spectral density function, $\hat{f}\left(\lambda \right)$, and plugging it into the expression above. There are several methods to estimate the spectral density of a Gaussian process, and hence produce an estimator of the entropy rate. Note that we can use this framework even in the cases of discrete-valued, discrete-time processes, using sampling techniques which can be used to calculate the integral in Equation (1). A variety of parametric and non-parametric techniques have been developed to estimate the spectral density of a Gaussian process. We will refer to these as either parametric/non-parametric or parametric/parametric for the classification by their entropy estimate and modelling estimate type.

#### 3.1.1. Maximum Entropy Spectral Estimation

A common technique used for the inference of spectral density is maximum entropy spectral estimation. It is a fitting paradigm where the best estimate is considered to be the estimate that maximises the entropy, that is, has the highest uncertainty. This paradigm is often called the Principle of Maximum Entropy, introduced by Jaynes [63].

These techniques were introduced by Burg [7,8], when aiming to model seismic signals by fitting stochastic models. He showed that given a finite set of covariance constraints for a process $\left\{X_{i}\right\}$, $E\left[X_{i}X_{i+k}\right]=\alpha_{k},\;k=0,1,\ldots ,p,$ then, the process that is the best fit for the constraints, given a maximum entropy approach, is the class of autoregressive processes, AR(p),

$$\begin{array}{cc} X_{n} & =-\sum_{k=1}^{p}a_{k}X_{n-k}+\epsilon_{n}, \end{array}$$

where $\epsilon_{n}∼N\left(0,\sigma^{2}\right)$ is normally distributed and $a_{k},\sigma^{2}$ are selected to fit the constraints [64].

This type of analysis can be generalised to auto-regressive moving-average, ARMA(p,q), models of the form

$$\begin{array}{cc} X_{n} & =-\sum_{k=1}^{p}a_{k}X_{n-k}+\sum_{k=1}^{q}b_{k}\epsilon_{n-k}, \end{array}$$

where the additional parameters, $b_{k}$, are selected to fit the behaviour of the noise process. Maximum entropy spectral analysis in this case also has to consider the function of the noise, called the impulse response function. It was shown by Franke [65,66] that ARMA is the maximum entropy process given a finite set of constraints on the covariances and on the impulse response function, $E\left[X_{i}\epsilon_{i-k}\right]=\sigma_{\epsilon }^{2}h_{k},k=1,\ldots ,q,$ where $\sigma_{\epsilon }^{2}$ is the variance of the noise variables and $h_{k}$ are the parameters of the impulse responses.

The entropy rate of the AR(p) and ARMA(p,q) classes of processes does not need to perform the integration over the spectral density function as the rate is known to be [67]

$$\begin{array}{c} h\left(\chi \right)=\frac{1}{2}\log\left(2\pi e\sigma_{\epsilon }^{2}\right). \end{array}$$

That is, the new information at each step of the process arises purely from the innovations, and if we can estimate the variance of the innovations, then we can infer the entropy rate directly. This has been extended to the ARFIMA(p,d,q) class of processes, where a process passed through a linear filter ${\left(1-L\right)}^{d},-\frac{1}{2}<d<\frac{1}{2}$, of the lag parameter *L*, i.e., $LX_{n}=X_{n-1}$ is an ARMA(p,q) process, with the same entropy rate [67]. However, for a fixed process variance, the entropy rate in this case is dependent upon the fractional parameter, *d*.

#### 3.1.2. Maximum Likelihood Spectral Estimation

In contrast to maximum entropy techniques, there are a class of techniques using a likelihood-based approach. This selects model parameters based on a likelihood function, which is the probability of parameters that would have generated the observations. In contrast to maximum entropy techniques, maximum likelihood requires the assumption of a model of the data, from which the likelihood function is calculated. The maximum entropy technique gives the best class of models which maximise the entropy, given some observed constraints.

These were first developed by Capon [9], to estimate the power spectrum from an array of sensors. Each sensor’s signal is modelled as $x_{i}=s+n_{i}$, where $x_{i}$ is the observed value at a sensor *i*, *s* is the signal and $n_{i}$ is the noise at sensor *i*. The maximum likelihood assumption is used in the density of the noise, a multivariate normal distribution, and then, a maximum likelihood estimate is made for the underlying signal.

Connections between the maximum entropy and maximum likelihood paradigms have been found in some aspects of spectral estimation. Landau [68] makes a connection between the maximum likelihood estimate of a spectral measure based on a one-parameter distribution and the maximum entropy spectral measure, where the maximum entropy measure is the uniform average over all of the maximum likelihood spectral measures. In the one-parameter case, maximum entropy is the uniform average over the parameters of the maximum likelihood estimators.

These approaches can then be used for an entropy rate estimate by calculating Equation (1) above, by plugging in the inferred spectral density function.

#### 3.1.3. Non-Parametric Spectral Density Estimation

The spectral density of a Gaussian process can be estimated directly without additional modelling assumptions, and then used in Equation (1) to estimate the entropy rate.

A common technique to estimate the spectral density is called the periodogram, which uses the fact that the spectral density is the Fourier transform of the autocorrelation function. Therefore, we can calculate the plug-in estimate of the spectral density estimate as

$$\begin{array}{c} \hat{f}\left(\lambda \right)=\sum_{j=-\infty }^{\infty }\hat{\gamma (j)}e^{ij\lambda }d\lambda , \end{array}$$

where the autocorrelation function can be estimated from observed data as

$$\begin{array}{c} \hat{\gamma }\left(k\right)=\frac{1}{n-k}\sum_{i=1}^{n-k}X_{i}X_{i+k}. \end{array}$$

However, this can cause issues as it may not converge for large sample sizes. This motivated the research of the maximum entropy processes, given different autocorrelation constraints [8].

Some important work in the development of the periodogram on time series data is from Bartlett [27,28] and Parzen [31,32] showing the consistency of the periodogram. Smoothing techniques have been developed and expanded in work by Tukey [29] and Grenander [30].

Other techniques for non-parametric spectral density have been developed. Some examples, including Stoica and Sundin [33], consider the estimation as an approximation to maximum likelihood estimation. Other non-parametric techniques are robust to data from long memory processes, which have a pole at the origin of the spectral density, as shown by Kim [34]. Finally numerous bayesian techniques have been developed for smoothing [35], parametric inference of the periodogram [36,37], robust to long memory [38], using MCMC to sample a posterior distribution [39,40] and using Gaussian process priors [41,42,43].

### 3.2. Markov Processes

Markov processes have been used to model information sources since Shannon’s introduction of information theory [1]. In this section, we discuss entropy rate estimation assuming the Markov property, that is for a process ${\left\{X_{i}\right\}}_{i\in N}$,

$$\begin{array}{c} Pr\left(X_{n}=x_{n}|X_{n-1}=x_{n-1},\ldots ,X_{1}=x_{1}\right)=Pr\left(X_{n}=x_{n}|X_{n-1}=x_{n-1}\right). \end{array}$$

There are two main types of Markov processes considered. Firstly, a simple Markov chain, and secondly, hidden Markov models (HMM). We mention Markov jump processes at the end, which have had substantially less attention.

#### 3.2.1. Markov Chains

The entropy rate of a stationary Markov chain with state space, Ω, is given by

(2) $$H\left(\chi \right)=\sum_{i\in \Omega }\sum_{j\in \Omega }\pi_{i}p_{ij}\log p_{ij},$$

where the $p_{ij}=Pr\left(X_{n}=J|X_{n-1}=i\right)$ form the probability transition matrix and $\pi_{i}$ is the stationary distribution for the Markov chain ([62], Theorem 4.2.4). For this approach, an implicit assumption of an ergodic Markov chain is required, for the existence of the stationary distribution. A few different approaches have been developed to estimate this quantity, which utilise parametric or non-parametric estimators.

The approach that has received most attention is to estimate the stationary distribution, and the probability transition matrix directly, which was inspired by the description of plug-in estimators for single samples by Basharin [10]. Maximum likelihood estimation techniques have been developed by Ciuperca and Girardin [11], on a finite state space, Girardin and Sesboue [55,56] on two state chains, and Ciuperca and Girardin [12] on countable state spaces. These utilise maximum likelihood estimators for $\pi_{i}$ and $p_{ij}$, given observations of the chain $X=\left(X_{0},\ldots ,X_{n}\right)$, with state space E=1,…,s, of the form

$$\begin{array}{cc} {\hat{p}}_{ij} & =\frac{N_{ij}\left[0,n\right)}{N_{i}\left[0,n\right)},\\ \mathrm{and},\; & \\ \hat{\pi_{i}} & =\frac{N_{i}\left[0,n\right)}{n}, \end{array}$$

where

$$\begin{array}{cc} N_{ij}\left[0,n\right) & =\sum_{m=1}^{n}{𝟙}_{\left\{X_{m}=j,X_{m-1}=i\right\}},\\ \mathrm{and},\; & \\ \;N_{i}\left[0,n\right) & =\sum_{j\in \Omega }N_{ij}\left[0,n\right)=\sum_{m=0}^{n-1}{𝟙}_{\left\{X_{m}=i\right\}}, \end{array}$$

are the counting functions of transitions from *i* to *j* and visits to *i*, respectively.

Whether estimating from one long sample or many groups of samples, the estimator from plugging these values into the entropy rate Equation (2) are strongly consistent and asymptotically normal [11,12,56]. For the countable case, for any finite sample, there will be transitions that have not been observed which are then set to 0, i.e., $p_{ij}=0$ if $N_{ij}\left[0,n\right)=0$; however, in the limit as n→0, the entropy rate still converges. These results have been extended to more general measures using extensions of the entropy rate, such as the Renyi Entropy [13].

Kamath and Verdu [14] have analysed the convergence rates for finite samples and single paths of estimators of this type. They showed that convergence of the entropy rate estimators can be bounded using the convergence rate of the Markov chain and the number of data observed.

A similar technique on finite state Markov chains was introduced by Han et al. [15], by enforcing a reversibility condition on the Markov chain transitions, in particular $\pi_{i}p_{ij}=\pi_{j}p_{ji}$. Using the stationarity of the transition function of the Markov chain, they define an estimator by utilising Shannon entropy estimators of the conditional entropy $H(X_{2}|X_{1}=i)$, and then, the estimator is

$$\begin{array}{cc} \hat{H} & =\sum_{i\in E}\hat{\pi_{i}}\hat{H}\left(X_{2}|X_{1}=i\right), \end{array}$$

where $\hat{\pi_{i}}$ is the stationary distribution estimate.

A similar approach was proposed by Chang [16] on finite Markov chains with knowledge of the probability transition matrices. It makes estimates using this knowledge by an entropy rate estimate using the observation $x_{k}$ at time *k*,

$$\begin{array}{cc} {\hat{H}}_{N} & =\frac{-\sum_{n=0}^{N-1}H\left(X_{n}\right)}{N}, \end{array}$$

given an initial state, $X_{0}=x$ and where $H(X_{n})$ is the Shannon entropy given knowledge of the current state, $X_{n}\in \Omega$. This is the same as using the maximum likelihood estimator of $\pi_{i}$, considering the probabilities as parameters, and then having a known conditional entropy estimate, as in the previous approach by Han et al. [15]. Chang was able to show that there is an exponential rate of convergence of this technique to the real value [16]. A similar result is obtained by Yari and Nikooravesh [17], showing an exponential convergence rate for this type of estimator under an assumption of ergodicity.

A final approach by Strelioff et al. [18] utilises Bayesian techniques to calculate the entropy rate of a Markov chain, using the connection to statistical mechanics. The model parameters, the probability transitions of the *k*th-order Markov chain are inferred as a posterior using a prior distribution, incorporating observed evidence. This is formulated as

$$\begin{array}{cc} Pr\left(\theta_{k}|M_{k}\right)Pr\left(D|\theta_{k},M_{k}\right) & =Pr(D,\theta_{k}|M_{k}), \end{array}$$

where *D* is the data, $M_{k}$ is a *k*th order Markov chain and $\theta_{k}$ are the parameters, transition probabilities of the Markov chain. The same framework can be applied to other information theoretic measures, such as the Kullback–Leibler divergence.

#### 3.2.2. Hidden Markov Models

A generalisation of Markov chains is given by hidden Markov Models, where we observe a sequence, ${\left\{Y_{i}\right\}}_{i\in Z^{+}}$ where there is an underlying Markov chain, ${\left\{X_{i}\right\}}_{i\in Z^{+}}$, and the probabilities of the observations of the hidden Markov model only depend on the current state of the Markov chain,

$$\begin{array}{c} Pr\left(Y_{n}=y_{n}|Y_{n-1}=y_{n-1},\ldots ,Y_{1}=y_{1},X_{n}=x_{n},\ldots ,X_{1}=x_{1}\right)=Pr\left(Y_{n}=y_{n}|X_{n}=x_{n}\right). \end{array}$$

Hence, this also exhibits the Markov property with dependence on the latent Markov chain.

In general, there is no known expression to directly calculate the entropy rate of a hidden Markov model [69,70,71,72], so we cannot just describe the techniques with respect to a plug-in expression for this class of models. However, some upper and lower bounds have been given by Cover and Thomas ([62], p. 69), and a proof of convergence of the bounds to the true value. It was shown that the entropy rate function is analytic in its parameters in [73], and it has been shown that the entropy rate function of a hidden Markov model varies analytically in its parameters, with some assumptions on the positivity of the transition matrix of the embedded Markov chain.

In the more specific case of binary-valued models, where both the Markov chain ${\left\{X_{i}\right\}}_{i\in Z^{+}}$ and observed random variables ${\left\{Y_{i}\right\}}_{i\in Z^{+}}$ are binary-valued, there have been expressions derived based on a noise model using a series expansion and analysing the asymptotics [74,75,76], and some analysis which links the entropy rate to the Lyapunov exponents, arising in dynamical systems [70]. Nair et al. [19] generated some upper and lower bounds, depending on the stationary distribution of the Markov chain and the entropy of a Bernoulli random variable. Lower bounds were further refined by Ordentlich [21], by creating an inequality that utilises a related geometrically distributed random variable. The exact expression remains elusive and is an active topic of research; however, as pointed out by Jacquet et al. [70], the link with Lyapunov exponents highlights the difficulty of this problem in general.

Although there are no explicit estimators for HMMs, Ordentlich and Weissman [20] created an estimator for the binary sequence ${\left\{Y_{i}\right\}}_{i\in Z^{+}}$,

$$\begin{array}{cc} H(Y) & =E\left[H\left(\frac{e^{Y_{i}}}{1+e^{Y_{i}}}⋆p⋆\delta \right)\right], \end{array}$$

where ⋆ is the binary convolution operator and *p* and δ are the probability of the embedded Markov chain changing state and the probability of observing a different state from the Markov chain. Given these simplifications, we can obtain an expression in terms of the expectation of the random variable and the stationary distribution. Luo and Guo [22] utilised a fixed point expression that can be developed on the cumulative distribution function. Then, a conditional entropy expression is exploited to calculate an entropy rate estimate,

$$\begin{array}{cc} H(X_{1}|X_{0},Y_{-\infty }^{1}) & =E\left[H_{2}\left({\left(1+e^{-\alpha X_{0}-r\left(Y_{1}\right)-L_{2}}\right)}^{-1}\right)\right], \end{array}$$

where

$$\begin{array}{c} H_{2}\left(p\right)=-p\log_{2}p-\left(1-p\right)\log_{2}\left(1-p\right), \end{array}$$

is the binary entropy function,

$$\begin{array}{c} \alpha =\log\left(\left(1-\epsilon \right)/\epsilon \right),\\ r\left(y\right)=\log\frac{Pr_{Y|X}\left(y+1\right)}{Pr_{Y|X}\left(y-1\right)}, \end{array}$$

$Pr_{Y|X}\left(\right)$ is the conditional probability of random variable *Y* given *X* and $L_{2}$ is the log-likelihood ratio. Then, they computed this numerically to form estimates, using a technique that exploits the fixed-point structure in a set of functional equations.

Gao et al. [23] use a non-parametric approach using limit theorems discussed in Section 4.1, which is applied to other processes such as Markov chains. However, with some assumptions, results can be achieved using limit theorems and fitting parameters to data. Travers [24] uses a path-mergability condition, if there exist paths that emit a symbol from the process ${\left\{Y_{i}\right\}}_{i\in Z^{+}}$,

$$\begin{array}{cc} \delta_{i}\left(w\right) & =\left\{j\in E:Pr_{i}\left(X_{0}^{|w|-1}=w,Y_{\left|w\right|}=j\right)\right\} \end{array}$$

such that for two distinct states *i* and *j*, there is a state *k* that can be reached from both states while creating the same path, i.e.,

$$\begin{array}{c} k\in \delta_{i}\left(w\right)\cap \delta_{j}\left(w\right). \end{array}$$

Then, entropy rate estimates are made non-parametrically of the conditional entropy,

$$\begin{array}{cc} H_{T}\left(\chi \right) & =H(X_{T}|X_{T-1},\ldots ,X_{1}), \end{array}$$

which under the stationarity assumption converges to the entropy rate. Given these assumptions, the estimates converge to the true value in the total variation norm at an exponential rate.

Peres and Quas [25] then tackle the problem of finite-state hidden Markov models with rare transitions. Their analysis is performed by setting some rare transitions to 0. In this case, they have defined the entropy rate as the average over the possible paths *w*,

$$\begin{array}{cc} H(Y) & =\lim_{N\to \infty }\frac{1}{N}\sum_{w\in \Omega^{N}}Pr\left(Y_{1}^{N}=w\right)\log Pr\left(Y_{1}^{N}=w\right). \end{array}$$

Under these assumptions, some lower and upper bounds of the expression above were found. These bounds are composed of the sums of the entropy rate of the This was shown to be a consistent estimator of entropyMarkov chain alone, and the entropy of the conditional distribution of the observed variables given the latent Markov chain.

#### 3.2.3. Other Markov Processes

In addition Markov and hidden Markov chains, some less studied Markov processes have had parametric entropy rate estimators developed.

Dumitrescu [77] analysed Markov pure jump processes, which are processes that have an embedded discrete-time Markov chain with jumps occuring at random times, $T_{t}$, for the *t*th jump, where the rates are given by a generator matrix, $Q={\left(q_{ij}\right)}_{i,j\in \Omega }$. In this case, Dumitrescu [77] proved that the entropy rate is

(3) $$H\left(\chi \right)=\sum_{i\in \Omega }\pi_{i}\sum_{j\neq i}q_{ij}\log q_{ij}+\sum_{i\in \Omega }\pi_{i}\sum_{j\neq i}q_{ij},$$

for π, the stationary distribution of the Markov chain.

Regnault [26] showed that, similar to the results of Ciuperca and Girardin [11,12], the stationary distribution could be estimated consistently and is asymptotically normal, for both: one long sample path and an aggregation of multiple sample paths. Consistency and asymptotic normality of the generator matrix, $\hat{Q}$, also proved, which are estimated using

$$\begin{array}{cc} {\hat{q}}_{ij} & =\left\{\begin{array}{cc} \frac{N_{ij}\left[0,n\right)}{R_{i}\left[0,n\right)}, & \;\mathrm{if}\;R_{i}\left[0,n\right)\neq 0,\\ 0, & \;\mathrm{otherwise}, \end{array}\right. \end{array}$$

where $R_{i}\left[0,n\right)$ is the total time spent in state *i*. Regnault then proved that plugging these estimates into the parametric form of the entropy rate in Equation (3) results in consistent and asymptotically normal estimates of the entropy rate, for the case of estimation from one long single path and estimation of multiple paths.

### 3.3. Renewal/Point Processes

Another important class of stochastic processes are renewal processes, these processes are a sequence of independent realisations of an inter-event distribution. We define the renewal process $S={\left\{S_{i}\right\}}_{i\in N}$, where $S_{i}$ are the event times, and we define the inter-event times $X={\left\{X_{i}\right\}}_{i\in N}$, and note that $S_{i}=\sum_{j=0}^{i}X_{j}$. A key description of a renewal process is the counting function of events, which is defined similarly to the Markov chain case above, $N\left[0,n\right)=\sum_{j=0}^{\infty }{𝟙}_{\left\{S_{j}\leq n\right\}}$, where each jump increments N[0,n) by 1. The entropy rate in this case of discrete-time inter-event distribution is

$$\begin{array}{cc} H(S) & =\lambda H(X),\\ \; & =-\lambda \sum_{j=1}^{\infty }p_{j}\log p_{j}, \end{array}$$

where $\lambda =1/E[X_{1}]$.

Discrete renewal processes have been estimated by Gao et al. [23], for a discrete distribution of *X* being $p_{j},j=1,2,\ldots$, to model binary-valued time series. The estimator is simply,

$$\begin{array}{cc} H(S) & =-\hat{\lambda }\sum_{j=1}^{\infty }\hat{p_{j}}\log\hat{p_{j}}. \end{array}$$

This was shown to be a consistent estimator of entropy rate; however, in practise, it was shown that long strings can be undersampled, unless the process was observed for an extremely long time frame. This is another example of a non-parametric model inside of a parametric estimator. One could estimate $p_{j}$ parametrically, e.g., assume *X* is a geometric random variable, and then, $p_{j}=p{\left(1-p\right)}^{j}$ and then estimate the probability, the parameter *p*.

## 4. Non-Parametric Approaches

In this section, we will be discussing non-parametric estimators of the Shannon and differential entropy rate. In contrast to the previous section, the estimators presented here make very few assumptions about the form of the data generating process. However, there are still assumptions that are required to enable the analysis, in particular, the stationarity or ergodicity of the process, to allow for limit theorems which are used to develop estimators with the desired properties. Non-parametric methods are robust to the type of distribution and parameter choices of models ([78], p. 3). There has been more research interest for Shannon entropy rate estimation, rather than differential entropy rate. However, there has been considerable research into the estimation of differential entropy, see Beirlant et al. [79]. The interest into differential entropy estimation techniques continues, particularly with the increase in computational power, to enable efficient calculation of kernel-density-based techniques [80].

### 4.1. Discrete-Valued, Discrete-Time Entropy Rate Estimation

In this section, we will briefly describe some entropy rate estimators for discrete-valued, discrete-time processes. We will consider techniques that utilise completely non-parametric inference of quantities that can be used for entropy rate inference. Non-parametric estimators have a rich history in information theory as ways of characterising the complexity and uncertainty of sources of generating data, particularly when considering communication theory and dynamical systems.

The first estimator we discuss is based on the Lempel–Ziv compression algorithm [81]. The estimation technique is based on a limit theorem on the frequency of string matches of a given length, for each $n\in Z^{+}$. Given the length of the prefix sequences of a process starting at digit *i*, $x_{i},x_{i+1},\ldots ,$ we define,

$$\begin{array}{c} L_{i}^{n}\left(x\right)=\min\left\{L:x_{i}^{i+L-1}\neq x_{j}^{j+L-1},1\leq j\leq n,j\neq i\right\}, \end{array}$$

or the length of the shortest prefix of $x_{i},x_{i+1},\ldots$ which is not a prefix of any other $x_{j},x_{j+1},\ldots$ for j≤n. A limit theorem was developed by Wyner and Ziv [82], based on the string matching, which states,

$$\begin{array}{c} \lim_{n\to \infty }\frac{L_{i}^{n}\left(x\right)}{\log n}\to \frac{1}{h(\chi )},\;\mathrm{in}\;\mathrm{probability}. \end{array}$$

This was extended to almost sure convergence, a.s., by Ornstein and Weiss [83]. Utilising the idea of this theorem, estimation techniques were developed which utilise multiple substrings and average the $L_{i}^{n}$’s instead of estimating from one long string, to make accurate and robust estimates with faster convergence to the true value. The following statement by Grassberger [44] was suggested heuristically,

$$\begin{array}{c} \lim_{n\to \infty }\frac{\sum_{i=1}^{n}L_{i}^{n}\left(x\right)}{n\log n}=\frac{1}{h(\chi )},\;a.s. \end{array}$$

This expression was shown by Shields [84] to not hold except in the cases of simple dependency structures, such as independent and identically distributed (i.i.d.) processes and Markov chains. However, a weaker version does hold for general ergodic processes, which states that for a given ϵ>0, all but a fraction of at most ϵ of the $\sum_{i=1}^{n}L_{i}^{n}\left(x\right)/n\log n$, are within the same ϵ of 1/h(χ) [84].

This is converted to an estimation technique by taking a suitably large truncation, and calculating the above expression for 1/h(χ). However, to make consistent estimates for more complex dependency structures, where the limit expression above does not hold, additional conditions are required. Kontoyiannis and Suhov [85], and Quas [46] extended this concept to a wider range of processes, firstly to stationary ergodic processes that obey a Doeblin condition, i.e., there exists an integer r≥1 and a real number β∈(0,1) such that for all $x_{0}\in A,Pr\left(X_{0}=x_{0}|X_{-\infty }^{-r}\right)\leq \beta$, with probability one, and secondly, to processes with infinite alphabets and to random fields satisfying the Doeblin condition.

Kontoyiannis and Suhov [85] followed the results of Shields [84] and Ornstein and Weiss [83], to show that the above estimator is consistent in much further generality with the addition of the Doeblin condition. They also state that without the condition, 1/h(χ) is the asymptotic lower bound of the expression.

Another class of estimators, which was initially suggested by Dobrushin [86], uses a distance metric on the “closeness” of different strings. We let ρ be a metric on the sample space Ω, and define sequences of length *T*, as $X^{\left(i\right)}=\left(X_{i},X_{i+1},\ldots .,X_{i+T}\right)$, with each of the *n* sequences being independent. A nearest neighbour estimator is defined as,

$$\begin{array}{cc} \hat{h_{n}} & =-\frac{1}{n\log n}\sum_{j=1}^{n}\log\left(\min_{i:i\neq j}\rho \left(X^{\left(i\right)},X^{\left(j\right)}\right)\right). \end{array}$$

Grassberger suggested this as an estimator with the metric $\rho \left(x,y\right)=\max\left\{2^{-k}:x_{k}\neq y_{k}\right\}$ [44]. This is an equivalent formulation using the $L_{i}^{n}$ quantity, and therefore, the same results from Shields apply. Similar techniques for nearest neighbour estimation were developed by Kaltchenko et al. [47], and the convergence rate for the nearest neighbour estimator was shown by Kaltchenko and Timofeeva [48]. Another related estimator was developed by Vatutin and Mikhailov [49], where they calculated the bias and consistency for nearest neighbour estimation.

A generalisation of the nearest neighbour entropy estimator was introduced as a measure called Statentropy by Timofeev [50]. This estimator is defined as,

$$\begin{array}{cc} \hat{h_{n}} & =-\frac{1}{n\log n}\sum_{j=1}^{n}\log\left(\min_{i:i\neq j}^{\left(k\right)}\rho \left(X^{\left(i\right)},X^{\left(j\right)}\right)\right), \end{array}$$

where $\min^{\left(k\right)}$ is the *k*th order statistic, i.e., the *k*th smallest value of the pairwise comparisons. Hence, this is a generalisation of the nearest neighbour estimator, by considering the *k*th smallest value, rather than the minimum. This estimator has been shown to be consistent, with convergence rates to the entropy rate developed by Kaltchenko et al. [48].

### 4.2. Continuous-Valued, Discrete-Time Entropy Rate Estimation

A few techniques have been developed to provide differential entropy rate estimates for continuous-valued, discrete-time processes. There are two classes: relative measures, which can be used for comparison of complexity of a system, and absolute measures, which are intended to accurately estimate the value of differential entropy rate for a system.

The relative measures include two closely related approaches, approximate [51] and sample entropy [52], which utilise pairwise comparisons between substrings of realisations of the process to calculate a distance metric. Another popular approach is permutation entropy which utilises the frequency of different permutations of order statistics of the process [87], and then calculates the estimate using an analogue of Shannon entropy on the observed relative frequencies [53].

These techniques were developed to quantify the complexity of continuous-valued time series, and therefore, the intention is to compare time series as opposed to provide an absolute estimate. These types of measures from the dynamic systems literature have been successful in the analysis of signals to detect change [88,89,90]. From the probabilistic perspective, we have an interest in the accurate, non-parametric estimation of differential entropy rate from data, without any assumptions on the distribution of the underlying source, and to compare complexity using this quantity.

The final technique we consider, specific entropy [54], is an absolute measure of the entropy rate. Due to computational advances, the technique uses non-parametric kernel density estimation of the conditional probability density function, based on a finite past, and uses this as the basis of a plug-in estimator.

We present each of these techniques in more detail below.

#### 4.2.1. Approximate Entropy

Approximate entropy was introduced by Pincus [51], with the intention of classifying complex systems. However, it has been used to make entropy rate estimates, since it was shown in the original paper to converge to the true value in the cases of i.i.d. processes and first-order finite Markov chains. Given a sequence of data, $x_{1},x_{2},\ldots ,x_{N}$, we have parameters *m* and *r*, which represent the length of the substrings we use for comparison and the maximum distance, according to a distance metric, between substrings to be considered a match. Then, we create a sequence of substrings, $u_{1}=\left[x_{1},\ldots ,x_{m}\right],u_{2}=\left[x_{2},\ldots ,x_{m+1}\right],\ldots ,u_{N-m+1}=\left[x_{N-m+1},\ldots ,x_{N}\right]$ and we define a quantity,

$$\begin{array}{cc} C_{i}^{m}\left(r\right) & =\frac{1}{N-m+1}\sum_{j=1}^{N-m+1}{𝟙}_{\left\{d\left[u_{i},u_{j}\right]\leq r\right\}}, \end{array}$$

where d[x(i),x(j)] is a distance metric. Commonly used metrics for this measure are the $l_{\infty }$ and $l_{2}$ distances.

The following quantity, used in the calculation of the approximate entropy, is defined in Eckmann and Ruelle [91] and used in Pincus [51],

$$\begin{array}{cc} \Phi^{m}\left(r\right) & =\frac{1}{N-m+1}\sum_{i=1}^{N-m+1}\log C_{i}^{m}\left(r\right). \end{array}$$

We now define the approximate entropy, ApEn(m,r), which is,

$$\begin{array}{cc} ApEn(m,r) & =\lim_{N\to \infty }\left[\Phi^{m}\left(r\right)-\Phi^{m+1}\left(r\right)\right]. \end{array}$$

For finite sequences of length *N*, this is positively biased because of the logarithm function E[log(X)]≤log(E[X]) by Jensen’s inequality [92] and the counting of some substrings twice. The bias in this estimator decreases as the number of samples, *N*, becomes larger [92].

Pincus showed in his initial paper that approximate entropy would converge to the entropy rate for i.i.d and finite Markov chains [51]. However, this does not hold in more general cases. Approximate entropy is also quite sensitive to the two parameters, *m*, and *r*, and hence, care must be taken when selecting these parameters [92,93]. It is recommended that *m* has a relatively low value, e.g., 2 or 3, which will ensure that the conditional probabilities can be estimated reasonably well [92]. The recommended values for *r*, are in the range of 0.1σ−0.25σ, where σ is the standard deviation of the observed data [92]. Another approach has been suggested by Udhayakumar et al. [94] to replace *r* by a histogram estimator based on the number of bins, and generate an entropy profile based on multiple different *r*s, to reduce the sensitivity to this parameter.

#### 4.2.2. Sample Entropy

A closely related technique for estimating the entropy rate is sample entropy [52], which was developed to address the issues of bias and lack of relative consistency in approximate entropy. The sample entropy, SampEn, is a simpler algorithm than ApEn, with a lower time complexity to make an estimate and eliminating self-matches in the data.

We define sample entropy by using very similar objects to approximate entropy. Given a time series, $x_{1},\ldots ,x_{N}$, of length *N*, we calculate substrings $u_{i}^{m}=\left[x_{i},\ldots ,x_{i+m-1}\right]$ of length *m*, and choose the parameter, *r*, for the maximum threshold between strings. We now define two related quantities,

$$\begin{array}{cc} A & =\sum_{i=0}^{N-m}{𝟙}_{\left\{d\left[u_{i}^{m+1},u_{j}^{m+1}\right]<r\right\}},\\ B & =\sum_{i=0}^{N-m+1}{𝟙}_{\left\{d\left[u_{i}^{m},u_{j}^{m}\right]<r\right\}}, \end{array}$$

where $d[u_{i}^{m},u_{j}^{m}]$ is a distance metric, with the usual distance metrics $l_{\infty }$ and $l_{2}$. Finally, we define the sample entropy as,

$$\begin{array}{cc} SampEn & =-\log\frac{A}{B}. \end{array}$$

As *A* will be always less than or equal to *B*, this value will always be non-negative.

Sample entropy removes the bias that is introduced via the double counting of substrings in approximate entropy; however, sample entropy does not reduce the source of bias that is introduced by the correlation of the substrings used in the calculation [52,92].

#### 4.2.3. Permutation Entropy

In addition to these two related relative entropy rate estimation techniques, we introduce permutation entropy developed by Bandt and Pompe [53]. Unlike the previous two, this has not been shown to converge to the true entropy rate for particular stochastic processes. However, it was developed for the same purpose, to quantify the complexity of processes generating time series data. Further development of the theory was undertaken by Bandt and Pompe, justifying the development of permutation entropy as a complexity measure [87].

Given a set of discrete-time data, $x_{1},\ldots ,x_{N}$, we consider permutations, π∈Π of length *n* which represent the numerical order of the substring data. For example, with n=3, three consecutive data points $\left(2,7,5\right)$ and $\left(3,9,8\right)$ are examples of the permutation 021, and $\left(5,1,3\right)$ and $\left(7,4,5\right)$ are examples of the permutation 201, the numbers in the permutation represent the ordering of the substring. For every permutation π, the relative frequencies are calculated as

$$\begin{array}{cc} p(\pi ) & =\frac{\left|\{t|t\leq T-n,x_{t+1},...,x_{t+n}\;\mathrm{has}\;\mathrm{type}\;\pi |\right\}}{T-n+1}. \end{array}$$

Hence, we are working with approximations to the real probabilities; however, we could recover these by taking the limit as T→∞ by the Law of Large Numbers ([95], p. 73) using a characteristic function on observing the permutation, with a condition on the stationarity of the stochastic process.

The permutation entropy of a time series of order n≥2 is then defined as,

$$\begin{array}{cc} H(n) & =-\sum_{\pi \in \Pi }p\left(\pi \right)\log p\left(\pi \right). \end{array}$$

Permutation entropy has one parameter, the order *n*. The number of permutations for an order scales as n!, which creates a time complexity issue as the required computations grows very quickly in the size of the order. Hence, the minimum possible data required to observe all of the possible permutations of order *n*, is n! data. However, it is claimed that the permutation entropy is robust to the order of the permutations used [53]. In practise, smaller *n*s are used, such as n=3,4,5 due to the growth of the number of permutations which requires more data to observe all of the permutations [53].

#### 4.2.4. Specific Entropy

Specific entropy was defined by Darmon [54], to provide a differential entropy rate estimation technique that has a stronger statistical footing than the previously defined estimation techniques. The intent of the development of this quantity was to create a measure of the complexity of a continuous-valued, discrete-time time series, as a function of its state. Then, a differential entropy rate estimate is made by taking a time average of the specific entropy estimates. Therefore, it can be applied in particular to ergodic processes. The approach is to consider the short-term predictability of a sequence, by utilising a finite history of values to create a kernel density estimate of the conditional probability density function. Then, use the kernel density estimate to plug-in into the differential entropy rate formula, when using the conditional density function. For the calculation of this quantity, a parameter of the length of the history, *p*, is used in the kernel density estimation of the conditional probability density function.

The definition of the specific entropy rate makes a finite truncation of the conditional entropy version of the entropy rate, as follows from Theorem 1. One condition is required in the formulation of the theoretical basis, which is that the process being measured is conditionally stationary. That is, given the conditional distribution function of $X_{t+1}$, $\left(X_{t},\ldots ,X_{t-p+1}\right)=X$ does not depend on the value of *t* for a fixed length of history being considered, *p*. In the paper by Darmon [54], they showed that the conditional entropy up to order *p*, depends on the state specific entropy of a particular history $\left(x_{p},\ldots ,x_{1}\right)=x_{1}^{p}$ and the density of the possible pasts $\left(X_{p},\ldots ,X_{1}\right)=X_{1}^{p}$. This is shown by an argument which establishes that,

$$\begin{array}{c} h\left(X_{t}|X_{t-p}^{t-1}\right)=-E\left[E\left[\log f\left(X_{t}|X_{t-p}^{t-1}\right)\right]\right]. \end{array}$$

Given this relationship and the law of total expectation, the specific entropy rate of order *p*, $h_{t}^{\left(p\right)}$ is defined as

$$\begin{array}{cc} h_{t}^{\left(p\right)} & =h\left(X_{t}|X_{t-p}^{t-1}=x_{t-p}^{t-1}\right),\\ \; & =-E\left[\log f\left(X_{t}|X_{t-p}^{t-1}\right)\right],\\ \; & =-\int_{-\infty }^{\infty }f\left(x_{p+1}|x_{1}^{p}\right)\log f\left(x_{p+1}|x_{1}^{p}\right)dx_{p+1}. \end{array}$$

Hence, the specific entropy rate estimator, ${\hat{h}}_{t}^{\left(p\right)}$, defined by plugging in the estimate of the density obtained by kernel density estimation, $\hat{f}\left(x_{p+1}|x_{1}^{p}\right)$, is

$$\begin{array}{c} {\hat{h}}_{t}^{\left(p\right)}=-E\left[\log\hat{f}\left(x_{p+1}|x_{1}^{p}\right)\right]. \end{array}$$

Then, the estimate of the differential entropy rate of order *p*, $\hat{h^{\left(p\right)}}$, is defined as

$$\begin{array}{cc} {\hat{h}}^{\left(p\right)} & =\frac{1}{T-p}\sum_{t=p}^{T}{\hat{h}}_{t}^{\left(p\right)},\\ \; & =\frac{1}{T-p}\sum_{t=p}^{T}-E\left[\log\hat{f}\left(x_{p+1}|x_{1}^{p}\right)\right], \end{array}$$

which is the time average of all the specific entropy rates across the observed states.

Specific entropy relies on some parameters to construct the kernel density estimation, which is the length of the past, *p* and the p+1 bandwidths, $k_{1},\ldots ,k_{p+1}$ that are used in the kernel density estimation [54]. The parameter choice can have large impacts on the quality of the estimation, in particular depending on the length of the past used in the kernel density estimation. The suggested technique for selecting *p* is a cross-validation technique which removes an individual observation and *l* observations on either side. Then, the following expression is minimised for its parameters $p,k_{1},\ldots ,k_{p+1}$,

$$\begin{array}{cc} CV(p,k_{1},\ldots ,k_{p+1}) & =-\frac{1}{T-p}\sum_{t=p+1}^{T}\log{\hat{f}}_{-t:l}\left(X_{t}|X_{t-p}^{t-1}\right). \end{array}$$

where ${\hat{f}}_{-t:l}$ is the conditional density with the points removed [54]. A suggested approach is to take l=0 and only remove the individual observation [96]. In practise, it is advised to fix *p* and then calculate the bandwidths due to the computational complexity of the cross-validation [54].

## 5. Conclusions

The research on entropy rate estimation has been driven by the need to quantify the complexity and uncertainty of sources of data, for a wide range of applications, from communications to biological systems. There are still gaps in the research, with many potential parametric approaches that can be developed for different stochastic models, and improvements to existing non-parametric approaches. In particular, the development of non-parametric estimators for the differential entropy rate and the development of more efficient techniques for non-parametric estimation of the Shannon entropy rate. In addition, further research could be developed for more generalised entropy rates, such as the Fisher–Shannon [97] and Rényi [98] entropy rates.

## Figures and Tables

**Table 1:** Comparison of entropy rate estimation techniques into categories based on parametric/non-parametric techniques. The modelling estimate refers to the quantity that is estimated in the technique and the entropy rate estimate refers to the full entropy rate expression used. For example, if estimating entropy rate of a Markov chain using plug-in estimation. Then, the modelling estimates may be non-parametric for the transition probabilities, $p_{ij}$ and the stationary distribution, $\pi_{j}$. However, the entropy rate estimator is a parametric estimator for the Markov model. Hence, there are no non-parametric/parametric estimators because non-parametric entropy estimators do not use a model.

	Modelling Estimate	
Entropy Rate Estimate	Parametric	Non-Parametric
Parametric	[7,8,9,10,11,12,13,14,15,16,17,18,19,20,21,22,23,24,25,26]	[27,28,29,30,31,32,33,34,35,36,37,38,39,40,41,42,43]
Non-Parametric	N/A	[44,45,46,47,48,49,50,51,52,53,54]

**Table 2:** Comparison of entropy rate estimation techniques. They are partitioned into four categories based whether they are discrete or continuous time, and whether they work on discrete or continuous-valued data.

	Time	
State Space	Discrete	Continuous
Discrete	[10,11,12,13,14,15,16,17,18,19,20,21,22,23,24,25,26,44,45,46,47,48,49,53,55,56]	[23]
Continuous	[7,8,27,28,29,30,31,32,33,34,35,36,37,38,39,40,41,42,43,51,52,53,54]	N/A
